# Calibration of Electret-Based Integral Radon Monitors Using NIST Polyethylene-Encapsulated ^226^Ra/^222^Rn Emanation (PERE) Standards

**DOI:** 10.6028/jres.100.047

**Published:** 1995

**Authors:** R. Collé, P. Kotrappa, J. M. R. Hutchinson

**Affiliations:** National Institute of Standards and Technology, Gaithersburg, MD 20899-0001; Rad Elec Inc., 5714C Industry Lane, Frederick, MD 21701; National Institute of Standards and Technology, Gaithersburg, MD 20899-0001

**Keywords:** calibration, electret, emanation, environment, E-PERM^®^, ionization chamber, measurement, radium-226, radon-222, standards

## Abstract

The recently developed ^222^Rn emanation standards that are based on polyethylene-encapsulated ^226^Ra solutions were employed for a first field-measurement application test to demonstrate their efficacy in calibrating passive integral radon monitors. The performance of the capsules was evaluated with respect to the calibration needs of electret ionization chambers (E-PERM^®^, Rad Elec Inc.). The encapsulated standards emanate well-characterized and known quantities of ^222^Rn, and were used in two different-sized, relatively-small, accumulation vessels (about 3.6 L and 10 L) which also contained the deployed electret monitors under test. Calculated integral ^222^Rn activities from the capsules over various accumulation times were compared to the averaged electret responses. Evaluations were made with four encapsulated standards ranging in ^226^Ra activity from approximately 15 Bq to 540 Bq (with ^222^Rn emanation fractions of 0.888); over accumulation times from 1 d to 33 d; and with four different types of E-PERM detectors that were independently calibrated. The ratio of the electret chamber response *E*_Rn_ to the integral ^222^Rn activity *I*_Rn_ was constant (within statistical variations) over the variables of the specific capsule used, the accumulation volume, accumulation time, and detector type. The results clearly demonstrated the practicality and suitability of the encapsulated standards for providing a simple and readily-available calibration for those measurement applications. However, the mean ratio *E*_Rn_/*I*_Rn_ was approximately 0.91, suggesting a possible systematic bias in the extant E-PERM calibrations. This 9 % systematic difference was verified by an independent test of the E-PERM calibration based on measurements with the NIST radon-in-water standard generator.

## 1. Introduction

The National Institute of Standards and Technology (NIST) has over the past 3 to 4 years worked on the development of a new emanation standard for ^222^Rn measurement calibrations as described by Collé et al. [1]. This new standard is based on a polyethylene-encapsulated ^226^Ra solution that has been demonstrated to emanate a well-characterized and known quantity of ^222^Rn when employed in an “accumulation mode.” The encapsulated standard was intended to serve as a more convenient, easier-to-use, alternative to the conventionally employed ^226^Ra solution standards that have been disseminated by NIST for ^222^Rn emanation measurements for the past 40 or more years [2–4]. The latter standards were, of course, only certified for the ^226^Ra mass content or at later times (circa mid-1980s) the ^226^Ra radioactivity content. The new encapsulated standards that are certified in terms of two parameters, both the ^226^Ra activity and the ^222^Rn emanation fraction, have, of necessity, a larger overall calibration uncertainty. Nevertheless, it was envisaged that the encapsulated standards would be sufficiently accurate and efficacious for calibrating instruments used in a variety of measurement applications, particularly those involving routine screening and monitoring of indoor radon air quality.

This paper describes the first demonstration of an application of the use of these polyethylene-encapsulated-^226^Ra/^222^Rn-emanation (PERE) standards^1^ for the calibration of a routine monitoring technique and measurement method. It applies to passive integral measurements of average ^222^Rn concentrations in air with “E-PERM”^2^ electret ionization chambers. The use, characteristics and performance of these monitors has been previously and extensively described by Kotrappa et al. [7,8].

Use of Rad Elec Inc. (REI) electret chamber monitors is probably the most widely employed measurement technique in the United States for evaluating radon levels within buildings. The U.S. Environmental Protection Agency (EPA) administers and conducts a measurement proficiency program for commercial vendors of radon measurement services. EPA currently estimates (based on participation in the EPA proficiency program) that of the 600 or more such vendors who maintain their own primary “in house” measurement capability approximately 200 to 250, or at least 30 % to 40 %, utilized the REI E-PERM electret ionization chambers. The second-most widely employed measurement method in the EPA proficiency program is used by less than half this number. REI estimates that the method is used by 300 to 400 laboratories in 15 countries.

## 2. Experimental Methodology

The NIST encapsulated emanation standards [1] consist of right circular cylinders of polyethylene having a 0.32 cm outside diameter and a 1.0 cm effective length along the emanating surface (overall length is ≃ 2 cm), and are gravimetrically filled with ≃ 0.08 g of calibrated ^226^Ra solutions having a known activity concentration. The ends of the polyethylene tubes are stoppered with two 0.5 cm teflon plugs and are crimp sealed with stainless steel bands around the outer circumference.

The standards are certified by NIST in terms of two parameters that, when used in some type of suitable closed accumulation vessel, allow calculation of the ^222^Rn activity accumulated in the vessel after a given accumulation time. The two parameters are the total ^226^Ra activity *A*_Ra(r)_ contained in the capsule at some reference time, *t*_r_, and the ^222^Rn emanation fraction, *f* (i.e., the fraction of the total ^222^Rn generated by decay of ^226^Ra that is released from the capsule and contained within the volume of the accumulation vessel). Both parameters are calibrated in terms of measurements that can be directly related to the U.S. national radon measurement calibration standard (i.e., the pulse-ionization-chamber-based primary radon measurement system [3,9]) and to national and international radium standards maintained by NIST.

For a capsule having a ^226^Ra activity content of $A_{\text{Ra}}^{0}$ at the start of an accumulation (time *t* = 0) and a constant emanation fraction *f*, the growth of ^222^Rn activity as a function of time *t* within a closed accumulation vessel may be given in approximate form as

$$A_{\text{Rn}}=fA_{\text{Ra}}^{0}\left(1-e^{-\lambda_{\text{Rn}}t}\right)+A_{\text{Rn}}^{0}e^{-\lambda_{\text{Rn}}t}$$ (1)

since the ^226^Ra—^222^Rn genetic relationship satisfies the condition of radioactive secular equilibrium (i.e., *λ*_Rn_ ≫ *λ*_Ra_, where *λ*_Rn_ and *λ*_Ra_ are the decay constants for ^222^Rn and ^226^Ra, respectively). In Eq. (1), $A_{\text{Rn}}^{0}$ is the initially present ^222^Rn activity in the accumulation vessel (i.e., for the boundary condition *A*_Rn_ = $A_{\text{Rn}}^{0}$ at *t* = 0). The initial ^226^Ra activity $A_{\text{Ra}}^{0}$ is just given by $A_{\text{Ra}}^{0}$ = *A*_Ra(r)_ $e^{-\lambda_{\text{Ra}}T_{D}}$, where *T*_D_ = (*t*_0_ −*t*_r_) is the decay-time interval, and where all other terms were defined previously. When the encapsulated standards are used in an accumulation mode with integral detectors, e.g., the electret chambers used here, it is necessary to consider the total integral activity over the total accumulation or detector deployment time. The time integral of Eq. (1) gives the total integrated ^222^Rn activity *I*_Rn_ accumulated over some total accumulation time *T*_A_. Integrating from *t* = 0 to *t* = *T*_A_ yields

$$\begin{array}{r} \int_{0}^{T_{A}}A_{\text{Rn}}dt=I_{\text{Rn}}=fA_{\text{Ra}}^{0}\int_{0}^{T_{A}}\left(1-e^{-\lambda_{\text{Rn}}t}dt\right)\\ +A_{\text{Ra}}^{0}\int_{0}^{T_{A}}e^{-\lambda_{\text{Rn}}t}dt=fA_{\text{Ra}}^{0}\left[T_{A}-\left(\frac{1}{\lambda_{\text{Rn}}}\right)\left(1-e^{-\lambda_{\text{Rn}}T_{A}}\right)\right]\\ +A_{\text{Rn}}^{0}\left(\frac{1}{\lambda_{\text{Rn}}}\right)(1-e^{-\lambda_{\text{Rn}}T_{A}}). \end{array}$$ (2)

For the case of $A_{\text{Rn}}^{0}$ = 0 (i.e., no ^222^Rn activity initially present in the accumulation vessel), the integrated activity is just

$$I_{\text{Rn}}=fA_{\text{Ra}}^{0}\left[T_{A}-\left(\frac{1}{\lambda_{\text{Rn}}}\right)\left(1-e^{-\lambda_{\text{Rn}}T_{A}}\right)\right].$$ (3)

Alternatively, one may consider the time-averaged ^222^Rn activity *Ā*_Rn_ over the time interval *T*_A_ to be

$${\bar{A}}_{\text{Rn}}=\frac{\int_{0}^{T_{A}}A_{\text{Rn}}dt}{\int_{0}^{T_{A}}dt}=\frac{I_{\text{Rn}}}{T_{A}},$$

or

$${\bar{A}}_{\text{Rn}}=fA_{\text{Ra}}^{0}\left[1-\left(\frac{1}{\lambda_{\text{Rn}}T_{A}}\right)(1-e^{-\lambda_{\text{Rn}}T_{A}})\right]$$ (4)

for the simplified case of $A_{\text{Rn}}^{0}$ ≃ 0.

Four of the prototype encapsulated standards were used in this study. They ranged in ^226^Ra activity $A_{\text{Ra}}^{0}$ from approximately 15 Bq to 540 Bq (see Table 1). The uncertainty in $A_{\text{Ra}}^{0}$ for each capsule, in terms of a relative “expanded combined standard uncertainty” (i.e., a coverage factor *k* = 2 and thus a 2 standard deviation estimate [10,11]), was approximately $2u_{A_{\text{Ra}}^{0}}$ = 0.87 %. The ^222^Rn emanation fraction for the prototype capsules was determined [1] to be *f* = 0.888 with a relative expanded uncertainty of 2*u_f_* = 3.4 %.^3^

By normal conventions of the NIST Radioactivity Group, which for the most part are wholly compatible with those adopted by the principal international metrology standardization bodies [10,11], all individual uncertainty components are expressed in terms of experimental standard deviations (or experimental standard deviations of the mean where appropriate) or quantities assumed to correspond to standard deviations irrespective of the method used to evaluate their magnitude. All of these uncertainty components are designated as “standard uncertainties.” A propagated uncertainty, termed a “combined standard uncertainty,” is expressed as what is assumed to be an estimated standard deviation which is equal to the positive square root of the total variance obtained by summing all variance (square of the standard uncertainty) and covariance components, however evaluated, using the law of propagation of uncertainty for the specific mathematical function given by the model of the measurement procedure [10]. By recently established NIST policy [11], the combined standard uncertainty is multiplied by a “coverage factor” of *k* = 2 to obtain an “expanded uncertainty” which is assumed to provide an uncertainty interval having a level of confidence of roughly 90 % to 95 %. For comparative purposes, it should be noted that previous SRM certificates issued by the NIST Radioactivity Group used comparably-based uncertainty coverage factors of *k* = 3. This former practice was historically rooted and was assumed to provide certified uncertainty intervals with somewhat higher confidence levels, approaching 95 % to 99 %. The component uncertainties comprising $2u_{A_{\text{Ra}}^{0}}$ and 2*u_f_* may be found in Collé et al. [1].

Of interest here is the uncertainty in *A*_Rn_ and *I*_Rn_, which may be obtained by invoking the propagation of uncertainty “law” [10,11] to sum all component variances and covariances using the appropriate functional forms. The relative uncertainties in *A*_Rn_ and in *I*_Rn_ (for the simplified cases with $A_{\text{Rn}}^{0}$ ≃ 0) are, from Eqs. (1) and (3) (and assuming the variables *f*, $A_{\text{Ra}}^{0}$, *λ*_Rn_, and *T*_A_ were all uncorrelated),

$$2u_{A_{\text{Rn}}}=2\sqrt{u_{f}^{2}+u_{A_{\text{Ra}}^{0}}^{2}+{\left[\frac{\lambda_{\text{Rn}}T_{A}e^{-\lambda_{\text{Rn}}T_{A}}}{\left(1-e^{\lambda_{\text{Rn}}T_{A}}\right)}\right]}^{2}(u_{\lambda_{\text{Rn}}}^{2}+u_{T_{A}}^{2})}$$ (5)

and

$$2u_{I_{\text{Rn}}}=2\sqrt{u_{f}^{2}+u_{A_{\text{Ra}}^{0}}^{2}+{\left[\frac{T_{A}(1-e^{-\lambda_{\text{Rn}}T_{A}})}{T_{A}-\frac{1}{\lambda_{\text{Rn}}}(1-e^{-\lambda_{\text{Rn}}T_{A}})}\right]}^{2}u_{T_{A}}^{2}+{\left[\frac{1-e^{-\lambda_{\text{Rn}}T_{A}}\left(1+\frac{T_{A}}{\lambda_{\text{Rn}}}\right)}{T_{A}-\frac{1}{\lambda_{\text{Rn}}}(1-e^{-\lambda_{\text{Rn}}T_{A}})}\right]}^{2}u_{\lambda_{\text{Rn}}}^{2}}$$ (6)

where the standard uncertainties *u* in each case are assumed relative standard deviations (e.g., *u_x_* = *s_x_*/*x* for any variable *x*). Throughout this study, the magnitude of the uncertainties $u_{\lambda_{\text{Rn}}}$ ≃ 0.05 % and $u_{T_{A}}$ ≤ 0.01 %, even in propagation over long accumulation times of up to *T*_A_ = 33 d, are negligible in comparison to *u_f_* ≃ 1.7 % and $u_{A_{\text{Ra}}^{0}}$≃ 0.43 %. The quantities *f* and $A_{\text{Ra}}^{0}$ are, however, correlated. Their uncertainties include a common uncertainty component *u*_c_ due to a ^226^Ra calibration factor used in the determination of *f* and $A_{\text{Ra}}^{0}$. Its magnitude is roughly *u*_c_ ≃ 0.4 %. The estimated relative uncertainties^4^ in *A*_Rn_ [Eq. (1)] and *I*_Rn_ [Eq. (3)] are thus approximately

$$2u_{A_{\text{Rn}}}≃2u_{I_{\text{Rn}}}≃2\sqrt{u_{f}^{2}+u_{A_{\text{Ra}}^{0}}^{2}-u_{c}^{2}}≃3.4\%$$

The NIST encapsulated standards were used in accumulation vessels provided by REI. The majority of the accumulation measurements were performed with nominal “one U.S. gallon,” screw-capped and gasketed glass jars that are a component part of the REI radon-in-water measurement test kit [12,13]. The jar lids were further sealed with compression collars fabricated out of thick sleeves of natural rubber that were clamped with metal bands. The total volume of the jars was independently measured by REI and by NIST by filling them with known volumes of water, and the total accumulation volume after subtracting the excluded volume for the E-PERM chamber housings was determined to be *V*_A_ = 3.622 L in most cases (see Table 1). The NIST-determined total volume (3.871 L) agreed with that of REI (3.842 L) within 0.8 %. The uncertainty in the NIST-determined total volume was estimated to be *u_V_* ≃ 0.6 % and on consideration of the uncertainties in the volume excluded by the detectors, the estimated uncertainty in the accumulation volume was taken to be $u_{V_{A}}$ ≃ 1 %.

To investigate possible systematic accumulation volume effects or discrepancies that might have resulted from the arbitrary choice of accumulation volume, a second, larger accumulation vessel was used for a few experiments. This vessel was a nominal 10 L, commercially available,^5^ plastic vacuum desiccator that provided accumulation volumes *V*_A_ of about 9.8 L and 10.1 L when used in configurations with different numbers of deployed detectors. The manufacturer specified that it could hold a vacuum of 3.3 kPa (25 Torr) for up to 24 h, and thereby provided some assurance of the vessel’s integrity against leaks.

Depending on the needs for a particular measurement in terms of the average ^222^Rn activity concentration expected and the chosen detector deployment time, E-PERM detectors of three different chamber volumes and two different sensitivities are commercially available from REI. E-PERM chambers of nominal 50 mL volume (designated “L”) and nominal 210 mL volume (designated “S”), and electrets of both high sensitivity (designated “ST” for short-term deployment) and low sensitivity (designated “LT” for long-term deployment) were used in this work. This resulted in a combination of four detector types, designated SST, SLT, LST, and LLT for (i) large-volume chamber and high-sensitivity electret; (ii) large-volume chamber and low-sensitivity electret; (iii) small-volume chamber and high-sensitivity electret; and (iv) small-volume chamber and low-sensitivity electret, respectively. The E-PERM chambers were originally designed and fabricated so that the excluded volume of two L chamber housings (55 mL each) is approximately that of one S chamber housing (110 mL). Thus, nearly identical excluded volumes (and hence accumulation volumes *V*_A_) could be obtained using 2 SST (or 2 SLT) or 4 LLT (or 4 LST) detectors.

The detector response for an E-PERM electret chamber is a measured voltage change that is proportional to the ionization produced by the integral ^222^Rn activity concentration to which it is exposed. Each detector type has an independently determined calibration factor that relates the voltage change to an average ^222^Rn activity concentration *C*_E_. This calibration factor is not linear, but is a function of the electret voltage. Calibration details may be found in Kotrappa et al. [7,8]. For this study, the REI determined and reported average ^222^Rn activity concentrations *C*_E_ (as obtained from their electret measurements and independent calibrations) were converted into integral activity responses

$$E_{\text{Rn}}=C_{E}V_{A}T_{A},$$ (7)

which could be compared to the integral ^222^Rn activity *I*_Rn_ [Eqs. (2) or (3)] provided by the encapsulated emanation standards in an accumulation volume *V*_A_ over accumulation time interval *T*_A_.

Alternatively, the measured and reported values of the average ^222^Rn activity concentration *C*_E_ could be compared to *Ā*_Rn_/*V*_A_ [using *Ā*_Rn_ from Eq. (4)], which is mathematically equivalent to the comparison of *E*_Rn_ to *I*_Rn_ (i.e., *C*_E_/(*Ā*_Rn_/*V*_A_) = *E*_Rn_/*I*_Rn_ since *E*_Rn_ = *C*_E_ *V*_A_ *T*_A_ and *Ā*_Rn_ = *I*_Rn_/*T*_A_).

The uncertainty in *C*_E_, based on an uncertainty analysis by REI that is given as part of their routine measurement methodology, is approximately 5 % to 6 % for a relative 1 standard deviation uncertainty interval [8]. Comparatively, the uncertainties in *V*_A_ and *T*_A_ are almost negligible ( $u_{V_{A}}$ ≃ 1 % and $u_{T_{A}}$ ≤ 0.01 %) so that the uncertainty in *E*_Rn_, $u_{E_{\text{Rn}}}$, may be considered to be of a comparable 5 % to 6 % at a relative one standard deviation uncertainty interval.

Insofar as the E-PERM electret detectors operate as a type of ionization chamber, they are sensitive to environmental gamma-radiation exposure-rate fields and exhibit a corresponding background response. The routine measurement methodology used by REI for calculating *C*_E_ provides an appropriate gamma-radiation background correction [7,8]. The encapsulated ^226^Ra standards used in this work are not believed to have significantly increased the gamma-radiation background in the accumulation vessel above natural ambient levels. The magnitude of the effect can be approximated by considering the activity content of the capsule and typical capsule-to-detector geometry in the accumulation vessel. The exposure rate for a 500 Bq ^226^Ra point source at 10 cm can be expected [14] to be roughly 0.01 Gy·h^−1^ (1 µR·h^−1^) compared to gamma-radiation ambient levels of typically 0.1 Gy·h^−1^. Hence, the effect is estimated to be ⪞ 10 % of a relatively small correction [7,8].

The experimental configuration used for the accumulation trials consisted of suspending the encapsulated standard from a thin thread so that it was located about in the center of the accumulation vessel. The various deployed detectors were distributed, somewhat randomly, about the remaining accumulator volume. A minimum of two detectors were deployed for any given experimental trial. When two detectors were used, one was located above the encapsulated standard and the other was located nearly equidistantly below. When four or five of the smaller-volume electret chamber detectors were deployed, they were of necessity, located at varying distances from the capsule. As will be discussed subsequently, this somewhithered, haphazard detector placement had the result of randomizing effects due to possible ^222^Rn activity concentration gradients as a function of distance from the encapsulated standard.

The experimental protocol consisted of maintaining identical timing and capsule preconditioning before and between each accumulation trial. The encapsulated standards when not in use are stored in water-saturated air. Before each use, the capsules are “equilibrated” for a minimum of 24 h in an open space, i.e., in an infinite volume of air, so that the external ^222^Rn activity concentration approximates zero prior to their placement in the vessel and the start of an accumulation. A similar 24 h open-air equilibrium was performed between experimental trials so that each accumulation started under identical diffusion boundary conditions.

For each trial, an accumulation vessel with a preconditioned encapsulated standard and with the deployed detectors was sealed in the vessel’s ambient air at some chosen start time *t* = 0. The surface voltage of each detector’s electret was measured just preceding placement of the detector in the vessel. After the passage of a selected accumulation time interval *T*_A_, the vessel was opened and the detectors were removed. The electret voltage of each deployed detector was immediately remeasured, thereby permitting calculation of the integral average activity concentration *C*_E_ for each detector [7,8].

Inasmuch as the accumulation vessels were sealed with ambient air, the integrated activity concentrations *C*_E_ include the electret responses due to ambient ^222^Rn. This is the contribution due to the second terms for $A_{\text{Rn}}^{0}$ in both Eqs. (1) and (2). Given that the ambient ^222^Rn activity concentrations were typically less than 0.015 Bq·L^−1^, the effect is rather small. The ratio of the second to first terms in Eq. (2) is < 0.02 (or < 2 % of *I*_Rn_) for an accumulation time of 3 d for even the lowest-level 15 Bq encapsulated standard. At longer accumulation times the effect rapidly becomes negligible. The ambient contribution at shorter times down to *T*_A_ ≃ 1 d for the 15 Bq source is still less than 4 %.

The four detector types were deployed with the four encapsulated standards in the two different accumulation volumes over accumulation times ranging from 1 d to 33 d.

This study was conducted in the period September 1990 to May 1991 prior to the completion by NIST of the emanation fraction calibration and performance testing of the encapsulated emanation standards. The presented results, however, are based on the since-completed, final calibrations [1].

## 3. Results and Discussion

The results of 16 different accumulation trials are summarized in Table 1. The integral ^222^Rn activity in the accumulations ranged from about 1 Bq·d to over 1300 Bq·d. Values for the integral activities *I*_Rn_ provided by the NIST encapsulated standards were calculated from Eq. (3) using the tabulated values of $A_{\text{Ra}}^{0}$ and *T*_A_ with a well-determined (subsequently certified) emanation fraction of *f* = 0.888. Recall that the overall $2u_{I_{RN}}$ relative expanded uncertainty in *I*_Rn_ was estimated to be 3.4 %.

The E-PERM responses are tabulated as the mean *E*_Rn_ [Eq. (7)] averaged over the measurements on two to five electret-chamber detectors for each accumulation. The relative standard deviation of the mean, *s*_m_, in the E-PERM response for each accumulation was in the range of 0.5 % to 3.0 %, with one exception. As might be anticipated, the excepted *s*_m_ ≃ 8 % was obtained in the case of expected least precision, that for an integral ^222^Rn activity of ≃ 1 Bq·d acquired with the lowest-level 15 Bq ^226^Ra source in a short *T*_A_ = 1 d accumulation time interval. The REI estimated total uncertainty $u_{E_{\text{Rn}}}$ of 5 % to 6 % is well borne out by the observed *s*_m_ values.

Averaging over all 16 accumulations, the comparison ratio *E*_Rn_/*I*_Rn_ mean was 0.908 with a relative experimental standard deviation of the mean of 0.99 %. For any given single accumulation, the comparison of *E*_Rn_ to *I*_Rn_ can barely exclude the possibility of their equivalence on consideration of their respective total uncertainties. The two-sided uncertainty interval $\left((E_{\text{Rn}}/I_{\text{Rn}})\pm k_{\alpha }\sqrt{u_{E_{\text{Rn}}}^{2}+u_{I_{\text{Rn}}}^{2}}\right)$, obtained by propagating $u_{E_{\text{Rn}}}$ and $u_{I_{Rn}}$, overlaps unity at almost any confidence probability (*p*) level *α* ≲ 0.1 (i.e., ≳ 90 % confidence coefficient for *p* = 1 − *α*) with normalized variate *k_α_* ≳ 2. Yet, many of the component uncertainties, of which $u_{E_{\text{Rn}}}$ and $u_{I_{Rn}}$ are composed, are clearly fixed and common between accumulations. Therefore, one might expect that the propagated uncertainty $u_{E_{Rn}}/I_{Rn}$ ≳ 6.3 % would thereby overestimate the statistical variations expected to be observed from just the experimental condition replications. The considerably smaller observed *s*_m_ ≃1 % in the mean *E*_Rn_/*I*_Rn_ ratio is indicative of this. In addition, the data of Table 1 clearly show that the results for *E*_Rn_/*I*_Rn_ do not fluctuate about unity with a large statistical variation, but rather exhibit a definite systematic bias trend. In every accumulation case, *E*_Rn_ is systematically less than *I*_Rn_, with a range of 0.87 ≤ *E*_Rn_/*I*_Rn_ ≤ 0.99.

This systematic bias was further evidenced when the comparison ratios *E*_Rn_/*I*_Rn_ were analyzed and averaged across the four variables of the specific encapsulated standard used (Table 2), the accumulation volume (Table 3), accumulation time (Table 4), and detector type (Table 5). In all four cases, the mean ratios of *E*_Rn_/*I*_Rn_ within each variable subset of data were invariant within statistical variations compared to the overall mean for all 16 accumulation trials. Several examples can be used to illustrate this invariance. When averaging across the four capsules (Table 2), the mean *E*_Rn_/*I*_Rn_ ratio for any given capsule differed from the overall mean *E*_Rn_/*I*_Rn_ = 0.98 by −2.3 % (for CR-12) to + 2.8 % (for CR-4c). Similarly, when averaging across the six *T*_A_ time intervals (Table 4), the mean *E*_Rn_/*I*_Rn_ ratio for any given *T*_A_ differed from the overall mean by −3.1 % (for *T*_A_ = 33 d) to + 2.1 % (for *T*_A_ = 7 d), even though two of the six *T*_A_ values were based on single accumulation trials. When comparing the data for the two different accumulation vessels and their respective volumes (Table 3), the maximum range difference in the two means for *V*_A_ ≃ 3.6 L and *V*_A_ ≃ 10 L was ≃ 1.4 %. Again, averaging across the four detector types (Table 5), the mean ratio in *E*_Rn_/*I*_Rn_ for any given detector type differed from the overall mean by − 1.6 % (for SST) to + 3.5 % (for LST). Interestingly, but perhaps of no significance, is the observation that these two extremes were obtained with detectors having electrets of high sensitivity (“ST”). They were, however, also the averages obtained with the fewest number of accumulations. Lastly, one may observe that the grand averages of the means for each variable (last rows of Tables 2, 4, and 5) differ from the overall mean *E*_Rn_/*I*_Rn_ = 0.908 by less than 0.4 % of the overall mean in every case: the four capsule means (0.909) differ by + 0.11 %; the six *T*_A_ means (0.905) differ by − 0.33 %; and the four detector-type means (0.911) differ by + 0.33 %.

Additional statistical tests were performed on the data set. These included *χ*^2^-tests of the homogeneity in subsets of the observed sample variances (across the variables), *F*-tests of the homogeneity in the various subset sample means discussed above, *t*-tests of differences between the various means, and tests of possible variable correlations and biases using analysis of variance (ANOVA) techniques with sequential two-way classifications of the four variables. None of the tests indicated any statistically significant differences in any of the tested subset sample means or sample variances (although it must be mentioned that in many of the cases, the sensitivity and power of the test was low because of the small sample sizes and the small degrees of freedom).

One may, therefore, conclude that the mean comparison ratio *E*_Rn_/*I*_Rn_ ≃ 0.91 is a reasonably good indicator of the performance of the E-PERM electret chambers compared to the NIST encapsulated emanation standards when the latter are employed to obtain accumulated integral ^222^Rn activities. The comparison ratio was invariant across the four tested variables, and the confidence interval for the mean *E*_Rn_/*I*_Rn_ is *t_v_*_= 15_ *s*m = 2.9 % at a 99 % confidence level.

The observed invariance in *E*_Rn_/*I*_Rn_ across the four tested variables leads one to exclude several possible causes of biasing effects. This is important to consider, particularly in view of the observed average 9 % systematic difference between the E-PERM responses and the integral ^222^Rn activities provided by the NIST encapsulated standards.

One obvious possible bias effect, of course, is leakage loss of ^222^Rn from the accumulation vessel. This could occur as real ventilation leaks at the seals of the vessel lids or as losses of ^222^Rn diffusing into components of the vessels (e.g., the plastic lids or rubber gaskets with the 3.7 L jar, or the plastic 10 L accumulation vessel itself). The use of the two very different types of accumulation vessels having substantially different volumes and composition materials, and yet yielding virtually identical *E*_Rn_/*I*_Rn_ values, would seem to exclude this as a biasing effect. Similarly, any kind of leakage loss is not likely to be proportionately constant over the wide variation in accumulation times from *T*_A_ = 1 d to 33 d as was observed in the constant *E*_Rn_/*I*_Rn_ ratios over these intervals. A leakage loss can normally be considered to have a representative time constant *λ*_L_ such that the growth functions for ^222^Rn [e.g., Eq. (1)] would be modified by substituting an effective rate constant *λ* = *λ*_L_ + *λ*_Rn_ for the ^222^Rn decay constant *λ*_Rn_. The integral activity *I*_Rn_ given by Eq. (3) calculated with *λ* = *λ*_L_ + *λ*_Rn_ and calculated with just *λ*_Rn_ would not be proportionately constant in the two cases over widely different *T*_A_ intervals.

A second possible biasing effect could arise if the detectors were responding to lower ^222^Rn activity concentrations than that given by the time-averaged [Eq. (4)] concentration ${\bar{A}}_{Rn}/V_{A}$ because of concentration inhomogeneities and gradients within the accumulation vessel. Again, the results from several of the variable factors would tend to exclude this possibility. No attempt was made to provide any mixing in the accumulation vessels. Yet, the diffusion of radon in air is relatively rapid (with a Fick’s law diffusion coefficient on the order of 0.1 cm^2^ · s^−1^) compared to the *T*_A_ time intervals, and the effect of concentration gradients, particularly on integral measurements, can be expected to be very negligible. In addition, no discernable differences in the detector responses were seen for detectors deployed at varying distances from the encapsulated standard. One would expect that if concentration gradients caused a biasing effect, it would have been manifest in the data for the two substantially different accumulation volumes.

In the absence of any other plausible explanation for the average ≃ 9 % difference between the REI-measured E-PERM responses and the NIST-provided ^222^Rn integral activities, one is compelled to question the extant E-PERM calibration. To this end, an independent confirmatory test of the E-PERM calibration was performed in an attempt to verify the observed systematic difference. The test was based on REI measurements of ^222^Rn activity concentrations in water using their E-PERM system [12,13] compared to the NIST radon-in-water standard generator [16–18] and confirmatory 4πα liquid scintillation (LS) measurements. Details for this calibration verification test are provided in Appendix A. The results indicated that a comparison ratio of the average REI E-PERM responses compared to a NIST calibration was 0.936, with a combined relative standard deviation of the mean of *s*_m_ ≃ 2 % for the statistical sampling and measurement variations. This observed 6 % to 7 % difference is wholly compatible with the ≃ 9 % difference seen in the accumulation experiments with the PERE standards.^6^

Another interesting application of these standards was demonstrated previously [15]. They were used to experimentally determine elevation correction factors for radon monitors. If a radon monitor is calibrated at sea level and then used at another elevation, a correction is necessary because of differences in air density at the two elevations. The PERE standards were used to obtain known ^222^Rn concentration ratios in two accumulation vessels maintained at two different pressures. The ratio of the radon monitor responses in the two vessels establishes the effect of differing pressures which are relatable to different elevations.

## 4. A Concluding Note

This work provided a comparison between the extant REI calibration of their electret-ionization-chamber-based E-PERM systems and accumulated integral ^222^Rn activities obtained from the recently developed NIST polyethylene-encapsulated ^226^Ra/^222^Rn solution (PERE) emanation standards. The protocol and measurement methodology that was used could, of course, be invoked in a similar fashion to actually provide an independent and direct E-PERM calibration that could be related to U.S. national, and internationally recognized, ^226^Ra and ^222^Rn standards. The study, however, went beyond merely serving the interests and calibration needs for one particular ^222^Rn measurement method—even though the REI electret chamber monitors in terms of their wide-spread use for routine screening and monitoring of indoor radon air quality have a substantial importance. Much more significantly, this work clearly demonstrated the utility and efficacy of the encapsulated emanation standards for a much broader range of measurement applications beyond those previously investigated [1], namely those involving accumulated ^222^Rn activities for integral measurements.

## Figures and Tables

**Table 1:** Results of 16 accumulation trials comparing E-PERM electret chamber responses to integral ^222^Rn activities provided by NIST encapsulated-^226^Ra standards

Capsule identification	^226^Ra activity in capsule $A_{\text{Ra}}^{0}$ (Bq)	Accumulation volume *V*_A_ (L)	Accumulation time *T*_A_ (d)	Detector type (and number deployed)	E-PERM response		Integral ^222^Rn activity *I*_Rn_ (Bq·d)	Ratio *E*_Rn_/*I*_Rn_
					Mean *E*_Rn_ (Bq·d)	*s*_m_ (%)		
CR-10	14.80	3.622	1.000	SST (2)	1.005	8.0	1.122	0.896
		3.622	3.000	SST (2)	8.421	2.6	9.013	0.934
		3.622	7.000	SLT (2)	39.32	1.2	39.93	0.985
		3.622	14.00	SLT (2)	106.4	1.0	117.2	0.908
		3.567	33.00	LLT (5)	318.5	1.3	361.4	0.881
CR-21	147.4	3.622	1.000	SST (2)	9.515	2.1	11.18	0.851
		3.622	3.000	SLT (2)	85.72	1.0	89.78	0.955
		3.622	7.000	LLT (4)	349.3	3.0	397.2	0.879
		3.622	14.00	LLT (4)	1033.	1.8	1168.	0.884
CR-12	514.8	3.622	1.000	SLT (2)	33.88	2.6	39.04	0.868
		3.622	3.000	SLT (2)	280.2	1.5	313.6	0.893
		9.820	2.792	SLT (5)	242.9	–	277.5	0.875
		3.622	7.000	LLT (4)	1271.	0.85	1387.	0.916
CR-4c	543.8	10.15	1.000	LST (4)	38.51	0.76	41.24	0.934
		10.09_5_	2.667	LST (5)	252.1	0.47	266.7	0.945
		3.622	4.000	LLT (4)	513.4	1.4	557.5	0.921

**Table 2:** Analysis of the comparison ratio *E*_Rn_/*I*_Rn_ across the variable of the specific encapsulated standard used

Averaging across results with capsule	Number in mean	Ratio *E*_Rn_/*I*_Rn_	
		Mean	*s*_m_ (%)
CR-10	5	0.921	2.0
CR-21	4	0.892	2.5
CR-12	4	0.888	1.2
CR-4c	3	0.933	0.74
All capsules	16	0.908	0.99
Capsule means	4	0.909	1.2

**Table 3:** Analysis of the comparison ratio *E*_Rn_/*I*_Rn_ across the variable of accumulation volume used

Averaging across results with accumulation volume *V*_A_ (L)	Number in mean	Ratio *E*_Rn_/*I*_Rn_	
		Mean	*s*_m_ (%)
*V*_A_ ≃ 3.6	13	0.905	1.1
*V*_A_ ≃ 10.	3	0.918	2.4
All *V*_A_ values	16	0.908	0.99

**Table 4:** Analysis of the comparison ratio *E*_Rn_/*I*_Rn_ across the variable of accumulation time used

Averaging across results with accumulation time *T*_A_ (d)	Number in mean	Ratio *E*_Rn_/*I*_Rn_	
		Mean	*s*_m_ (%)
*T*_A_ = 1	4	0.887	2.0
*T*_A_ = 2.7 to 3	5	0.920	1.7
*T*_A_ = 4	1	(0.921)	
*T*_A_ = 7	3	0.927	3.4
*T*_A_ = 14	2	0.896	1.3
*T*_A_ = 33	1	(0.881)	
All *T*_A_ values	16	0.908	0.99
*T*_A_ means	6	0.905	0.89

**Table 5:** Analysis of the comparison ratio *E*_Rn_/*I*_Rn_ across the variable of detector type used

Averaging across results with detector type	Number in mean	Ratio *E*_Rn_/*I*_Rn_	
		Mean	*s*_m_ (%)
SST	3	0.894	2.7
SLT	6	0.914	2.1
LLT	5	0.896	1.0
LST	2	0.940	0.58
All detectors	16	0.908	0.99
Detector means	4	0.911	1.2

## Footnotes

1 The PERE standard is currently available as NIST Standard Reference Material SRM 4968 [5]. This paper was prepared in early 1993 and was withheld from submission for publication pending completion of the PERE standard calibration. The presently described study was performed with an earlier prototype version of SRM 4968. This prototype utilized mechanical compression seals, as described herein, which were found to be inadequate over long times. As a result, in the interim period following this study, a new heat-sealing procedure based on complete polyethylene encapsulation was developed for SRM 4968. A complete recalibration (and confirmation) of the previously obtained calibration factors for the compression-seal prototypes was also performed [6]. The mean emanation factor *f* obtained for the earlier prototype capsule used in this work and that for SRM 4968 is virtually indistinct (0.888 compared to 0.890). Subsequent electret measurements conducted during the period July–August 1993 with a set of 14 of the revised heat-sealed PERE standards confirmed the findings presented in this paper. Three independent trials with multiple replications of specific capsule, detector types, and accumulation times were used to perform this confirmation.

2 Certain commercial equipment, instruments, or materials are identified in this paper to foster understanding. Such identification does not imply recommendation or endorsement by the National Institute of Standards and Technology, nor does it imply that the materials or equipment identified are necessarily the best available for the purpose.

3 The PERE standards, issued as SRM 4968, were certified with slightly revised uncertainties of $2u_{A_{\text{Ra}}^{0}}$ = 0.93 % and 2*u_f_* = 4.0 %. The revisions arose from more exhaustive uncertainty component considerations in the recalibration [5,6].

4 Using revised uncertainty estimates for *u_f_* and $u_{A_{\text{Ra}}^{0}}$, as given for SRM 4968, the relative expanded uncertainties $2u_{A_{RN}}$ and $2u_{I_{RN}}$ are approximately 4.0 %.

5 Bel Art product # 42072, Bridgeport, New Jersey.

6 Calibration factors for the E-PERM detectors as provided by REI were revised in late 1994 to account, in part, for these observed systematic biases.

## 5. Appendix A. A Confirmatory Verification of the E-PERM Calibration Bias Based on ^222^Rn-in-Water Measurements

An independent confirmatory test of the extant E-PERM calibration was performed in an attempt to verify an observed ≃ 9 % systematic difference in the E-PERM integral ^222^Rn activity responses *E*_Rn_ [Eq. (7)] compared to the integral activities *I*_Rn_ [Eq. (3)] provided by NIST polyethylene-encapsulated ^226^Ra/^222^Rn emanation (PERE) standards. The test was based on REI measurements of ^222^Rn in water using their E-PERM system [12,13] compared to the NIST radon-in-water standard generator [16–18] and confirmatory 4π *α* liquid scintillation (LS) measurements.

The REI routine procedure for measuring dissolved ^222^Rn-in-water samples using their “Radon in Water Kit” with E-PERM electret chamber detectors has been described previously [12,13,19]. The procedure, in brief, consists of the following. A small water sample, appropriately collected and contained in a REI sample bottle, is placed in the bottom of a nominal “one U.S. gallon” glass accumulation jar. The protocol calls for efforts to minimize losses of ^222^Rn in transferring the sample. An E-PERM detector, with a pre-measured initial electret voltage, is suspended in the air space above the water sample. The lid of the jar is closed and sealed, and the jar is then gently agitated to aerate the water sample to accelerate the release of ^222^Rn into the air space. The methodology relies upon the constant partitioning of the ^222^Rn between the water and air contained within the accumulation vessel. At the conclusion of a desired accumulation time interval (typically of several days duration), the jar is opened to remove the E-PERM detector for measurement of the post-accumulation final electret voltage. The average ^222^Rn activity concentration in the air [identical *C*_E_ of Eq. (7)] is calculated by the REI conventional E-PERM procedure that is based on the accumulation time interval and initial and final electret voltage measurements using their provided calibration factors. The average ^222^Rn activity concentration in the water sample *C*_W_ is then derived from *C*_E_ using a simple partition model [11] that employs the necessary decay and accumulation time intervals, the relative volumes of water and air in the accumulation jar, and the Oswald partition coefficient.

For this confirmatory test of the E-PERM calibration, the NIST radon-in-water standard generator [16–18] was used to prepare calibrated water samples with known ^222^Rn concentrations. The generator utilizes an earlier prototype source of the polyethylene-encapsulated ^226^Ra solution in a small-volume accumulation chamber and with an ancillary mixing and dispensing system. It generates aqueous solutions of radium-free ^222^Rn of which multiple aliquots may be dispensed and used as standardized solutions for calibrating ^222^Rn-in-water assay procedures. As in all other NIST transfer standards for ^222^Rn, the generator calibration is directly relatable to the NIST primary ^222^Rn measurement system and in turn to national and international ^226^Ra standards [3].

The generator was used to prepare four samples in the REI sample bottles (nominal volume 68 mL) and six LS samples for NIST measurement. Each of the REI samples consisted of approximately 55 mL of blank, “radon-free,” distilled water (less than 0.003 Bq · g^−1 222^Rn); and 0.366 g of ^222^Rn solution containing *C*_Rn_ = 9.588 Bq · g^−1^ of ^222^Rn at the reference time (1336 DST on 26 April 1991). The 2*u*_Rn_ uncertainty in the total ^222^Rn activity in each sample (89.80 Bq total) is estimated to be about 2.3 % (cf., the uncertainty analyses in Hutchinson et al. [17] and Collé et al. [18]).

The REI samples were prepared by dispensing the ^222^Rn solution beneath the surface and at the bottom of the sample bottles which had previously been filled with the 55 mL of blank water. The samples were immediately transferred to REI for placement in their measurement jars. Possible losses of ^222^Rn from samples dispensed in this way was largely unknown, but based on previous REI experience and their measurement protocol it was thought to be minimal.

The six LS samples, each with an average 1.561 g of ^222^Rn solution as gravimetrically determined, were also dispensed into previously filled LS counting vials. These LS samples were interspersedly dispensed between the REI samples. The sample aliquots were dispensed in the sequence e1 : e2 : E1 : e3 : E2 : e4 : E3 : e5 : E4 : e6 where the lower-case e designator represents an LS sample and the upper-case E is an REI sample.

Two different LS cocktails were used for the measurements, viz., 18.8 g of “PCS” (a xylene-surfactant-based LS cocktail)^7^ and 20.0 g of “Ready Safe” (designated RS here, a polyarylalkane-surfactant-based cocktail).^8^ Matched blanks of nearly identical composition were also prepared for background subtractions. Each of the six LS samples along with the matched blanks were measured five times over 3 days.

The resultant ^222^Rn activity concentrations *C*_Rn_ for these samples were based exclusively on the present LS measurement assays, although they were also within 2 % agreement with the historical, canonical generator calibration factors [17,18]. The dispensed sample sizes for the REI samples were based on the known dispenser “mass divided by turn” calibration factor for the generator [17]. This factor was also reconfirmed in the present measurements from the gravimetrically-determined dispensed masses in the LS samples. The factor (0.7805 g per turn; *s*_m_ = 0.8 %) was within 1 % of the historical value—although for some inexplicable reason the dispensing precision was nearly a factor of 5 to 10 more variable than past history.

The measurement results for the six LS samples are given in Table A1. The reported value of *C*_Rn_ for each sample is the mean of 5 replicate measurements (corrected for ^222^Rn radioactive decay) obtained after the ^222^Rn and its short-lived daughters were in secular equilibrium. The precision of the measurements for each sample in terms of a relative experimental standard deviation of the mean *s*_m_ range from 0.13 % to 0.33 %. The Poisson statistical “counting error” for each of the 30 measurements ranged from 0.1 % to 0.2 %. The LS counting results were corrected for count-rate background from the matched blanks, ^222^Rn partitioning in the LS sample, dead-time losses, count-rate-versus-energy extrapolations to zero energy, and ^222^Rn radioactive decay to obtain the values of *C*_Rn_. The mean concentration *C*_Rn_ = 9.588 Bq · g^−1^ over the 6 LS samples has a relative experimental standard deviation of the mean of *s*_m_(*C*_Rn_) = 0.51 %. Analysis of the covariance matrix (for the five measurement by six sample matrix) and analysis of variance (ANOVA) calculations indicate no sampling or measurement biases in the calculated mean. The mean and its variance were invariant over the specific LS sample mean, over the five LS counting cycles, over the dispensed sample order, over the timing or sequence of the LS sample measurements, over the dispensed sample masses, and over the LS cocktail used. LS spectra also confirmed the absence of any ^226^Ra in the measured samples.

**Table A1:** NIST LS-measurement calibration results for the ^222^Rn-in-water samples

Sample	LS cocktail	Dispensed mass of ^222^Rn solution (g)	^222^Rn activity concentration *C*_Rn_	
			Mean (Bq·g^−1^)	*s*_m_ (%)
e1	PCS (18.79 g)	1.5787	9.649	0.22
e3		1.5331	9.536	0.27
e5		1.5421	9.707	0.18
e2	RS (20.05 g)	1.5191	9.616	0.23
e4		1.5862	9.645	0.13
e6		1.6070	9.373	0.33

	Mean *C*_Rn_		9.588	
	Number in mean		6	
	*s*_m_(*C*_Rn_) in %		0.51	

The mean total ^222^Rn activity from the E-PERM measurements on the four REI samples was 84.06 Bq with a relative experimental standard deviation of the mean of 1.7 %. The mean dispensed mass of the calibrated ^222^Rn solution in the samples was 9.366 g (with an estimated *s*_m_ = 0.8 %). The mean ^222^Rn activity concentration is then *C*_W_ = 8.975 Bq · g^−1^ with a combined relative experimental standard deviation of the mean *s*_m_(*C*_W_) = 1.9 %. The uncertainty in terms of a relative standard uncertainty was estimated by REI to be a canonical *u*_W_ = 5 % to 6 % based on their routine measurement protocol and experience [12,19].

The average E-PERM response compared to the NIST calibration can then be expressed as the comparison ratio

$$\frac{C_{W}}{C_{\text{Rn}}}=0.936$$

with a statistical sampling and measurement estimator of

$$\begin{array}{l} s_{m}=\sqrt{{\left[s_{m}(C_{W})\right]}^{2}+{\left[s_{m}(C_{\text{Rn}})\right]}^{2}}\\ \;≃2\% \end{array}$$

and a combined standard uncertainty in the *C*_W_/*C*_Rn_ ratio of approximately

$$\begin{array}{l} u=\sqrt{u_{W}^{2}+u_{\text{Rn}}^{2}}\\ \;≃6\% \end{array}$$

Expanding the confidence interval *C*_W_/*C*_Rn_ ± *k_α_u* for any normalized variate *k_α_* ≳ 2 with confidence coefficient level *p* ≥ 90 % (*α*= 1 − *p*/100 ≤ 0.1) cannot exclude the possibility that the “true” *C*_W_/*C*_Rn_ overlaps unity (and thereby the equivalence of *C*_W_ and *C*_Rn_ at that confidence level). Yet, the estimator of central tendency in this case, given by the mean *C*_W_/*C*_Rn_ = 0.936, is wholly compatible with the previously observed approximate − 9 % bias in the comparison of the averaged E-PERM responses to the integral ^222^Rn activities provided by the NIST PERE standards.
